# Hospitalizations, emergency room visits and causes of death in 198 patients with permanent hypoparathyroidism: a retrospective Austrian study (2005–2022)

**DOI:** 10.1530/EC-22-0533

**Published:** 2023-04-12

**Authors:** Julia Herteux, Simon Johannes Geiger, Christina Starchl, Johanna Windisch, Theresa Lerchl, Adelina Tmava-Berisha, Gerit Wünsch, Kathrin Eller, Astrid Fahrleitner-Pammer, Karin Amrein

**Affiliations:** 1 Medical University of Graz, Auenbruggerplatz, Graz, Austria

**Keywords:** hypoparathyroidism, hypocalcemia, renal disease, dialysis, kidney transplantation

## Abstract

**Objective:**

Chronic hypoparathyroidism (HP) is associated with acute and chronic complications, especially those related to hypocalcemia. We aimed to analyze details on hospital admissions and the reported deaths in affected patients.

**Design and methods:**

In a retrospective analysis, we reviewed the medical history of 198 patients diagnosed with chronic HP over a continuous period of up to 17 years at the Medical University Graz.

**Results:**

The mean age in our mostly female cohort (70.2%) was 62.6 ± 18.7 years. The etiology was predominantly postsurgical (84.8%). About 87.4% of patients received standard medication (oral calcium/vitamin D), 15 patients (7.6%) used rhPTH_1–84_/Natpar® and 10 patients (4.5%) had no/unknown medication. Two hundred and nineteen emergency room (ER) visits and 627 hospitalizations were documented among 149 patients, and 49 patients (24.7%) did not record any hospital admissions. According to symptoms and decreased serum calcium levels, 12% of ER (*n* = 26) visits and 7% of hospitalizations (*n* = 44) were likely attributable to HP. A subgroup of 13 patients (6.5%) received kidney transplants prior to the HP diagnosis. In eight of these patients, parathyroidectomy for tertiary renal hyperparathyroidism was the cause of permanent HP. The mortality was 7.8% (*n* = 12), and the causes of death appeared to be unrelated to HP. Although the awareness for HP was low, calcium levels were documented in 71% (*n* = 447) of hospitalizations.

**Conclusions:**

Acute symptoms directly related to HP did not represent the primary cause of ER visits. However, comorbidities (e.g. renal/cardiovascular diseases) associated with HP played a key role in hospitalizations and deaths.

**Significance statement:**

Hypoparathyroidism (HP) is the most common complication after anterior neck surgery. Yet, it remains underdiagnosed as well as undertreated, and the burden of disease and long-term complications are usually underestimated. There are few detailed data on emergency room (ER) visits hospitalizations and death in patients with chronic HP, although acute symptoms due to hypo-/hypercalcemia are easily detectable. We show that HP is not the primary cause for presentation but that hypocalcemia is a typical laboratory finding (when ordered) and thus may contribute to subjective symptoms. Patients often present with renal/cardiovascular/oncologic illness for which HP is known to be a contributing factor. A small but very special group (*n* = 13, 6.5%) are patients after kidney transplantations who showed a high ER hospitalization rate. Surprisingly, HP was never the cause for their frequent hospitalizations but rather the result of chronic kidney disease. The most frequent cause for HP in these patients was parathyroidectomy due to tertiary hyperparathyroidism. The causes of death in 12 patients appeared to be unrelated to HP, but we found a high prevalence of chronic organ damages/comorbidities related to it in this group. Less than 25% documented HP correctly in the discharge letters, which indicates a high potential for improvement.

## Introduction

### Background

Limited data exist on hospitalizations, emergency room (ER) visits and causes of death in chronic hypoparathyroidism (HP). HP is like a chameleon that can trigger diverse acute and chronic symptoms. The risk for comorbidities including renal, cardiovascular and infectious diseases appears to be increased in retrospective cohorts, with results of the ongoing PARADIGHM trial currently pending (1). Conflicting results also exist on the clinical significance of HP with regard to mortality, as some studies report a higher mortality in HP, while others do not find such a correlation (2, 3, 4).

Acute symptoms are most commonly triggered by hypocalcemia. They include muscle spasms, paresthesia, shortness of breath or even potentially life-threatening conditions like laryngospasm or arrhythmia (5, 6, 7). Long-term complications like chronic kidney disease (CKD), nephrolithiasis and neurocognitive dysfunction are also well-known complications of chronic HP (8, 9, 10).

In 2014, the Canadian National Hypoparathyroidism Registry started collecting medical data, including fracture risk, mortality and comorbidities (11). In a retrospective study design, they were able to evaluate the medical history of 130 patients (mean age 54 years) over a period of 6 years. Thirty four percent of all patients were hospitalized at least once because of chronic HP, and the nonsurgical subgroup contributed to more hospitalizations than the surgical one.

In 2020, Zanchetta *et al.* published a retrospective study analyzing the medical data of seven centers in Buenos Aires on the medical history of 322 HP patients (mean age 55 years) over the course of 33 years (12). Twenty-six percent of patients had at least one hospital admission due to hypocalcemia. The proportion of severe cases causing seizures was 4.3%, whereas the number of hospital admissions due to neuromuscular symptoms added up to 40.9%.

Cipriani *et al.* evaluated the medical data of 27,692 hospitalizations for HP (mean age 50 years) between 2006 and 2013 in a retrospective nationwide approach in Italy (13). The mean hospitalization rate for HP was 5.9/100,000 inhabitants per year, which is almost half the rate for primary hyperparathyroidism reported in a similar approach (14). Interestingly, there was a significant decrease for documented hospitalizations during the period 2006–2013.

### Objective

We aimed to collect new data on the causes of ER visits, hospitalizations and death in patients with chronic HP. Furthermore, we attempted to differentiate between HP-related causes, comorbidities and other causes. Lastly, we aimed to assess the awareness of hospital staff by documenting all diagnoses and orders of calcium tests.

## Materials and methods

### Study design and patients

**Figure 1:**
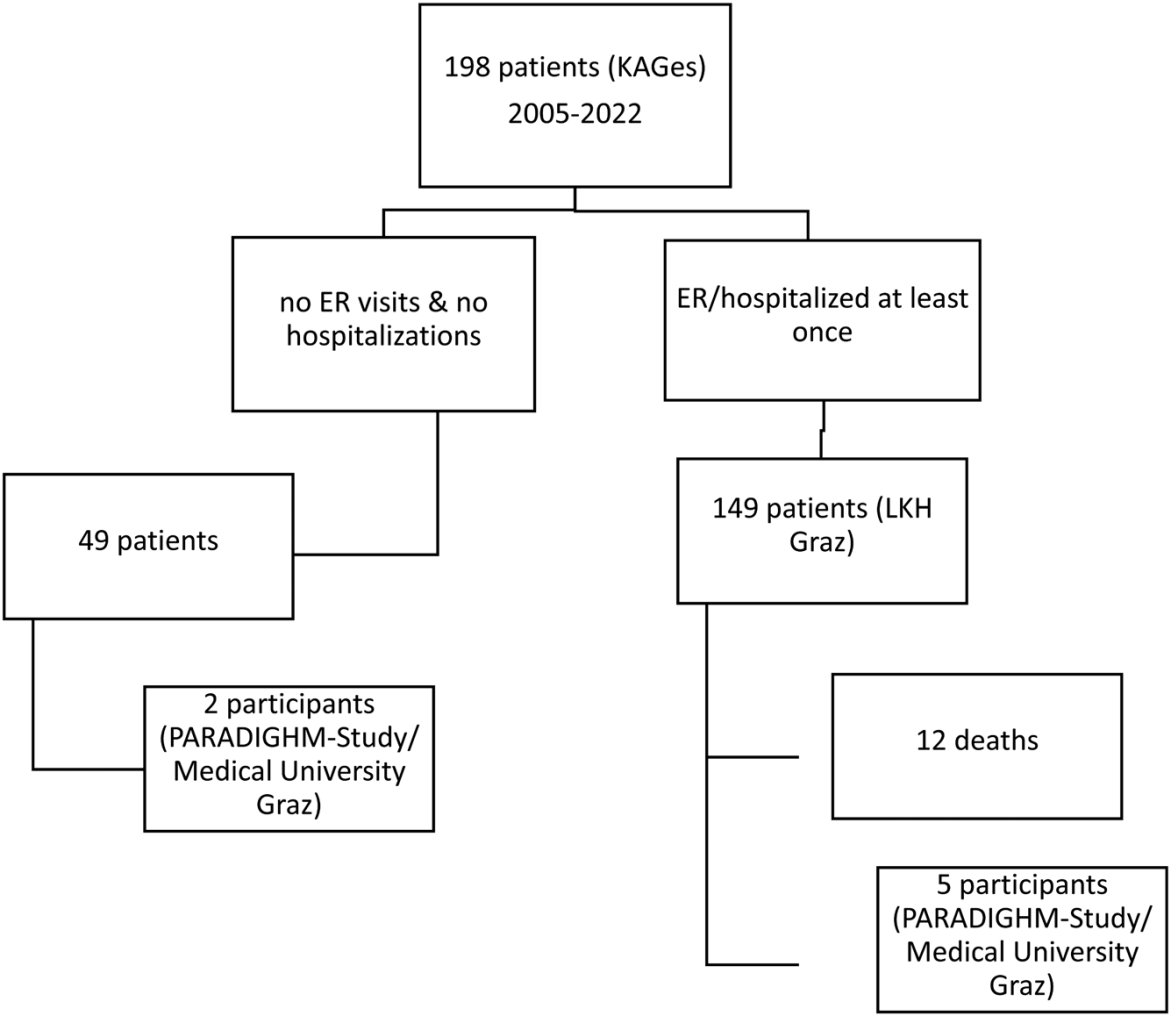
Flowchart of the study population.

In this retrospective study, we assessed the clinical information that was collected over the course of 17 years (since the introduction of MEDOCS, the patient data management system established in 2005 up to the final data collection in 2022). We identified a cohort of 198 patients with chronic HP, of whom 149 patients were hospitalized or presented at the ER, including seven participants of the PARADIGHM study (1). Data were only available for the Medical University Graz (Austria), the largest tertiary care center in the region (Fig. 1).

This study received approval from the ethics committee Graz in June 2022 (33-151 ex 20/21) and received permission of the University Clinic and KAGes to use their database for research purposes. The whole process was conducted in accordance with the principles of the Declaration of Helsinki. The participants were selected according to the following criteria: the documented diagnosis, parathyroid hormone (PTH) levels <30 pg/mL, hypo-/normocalcemia, with/without ongoing therapy, at least 2 recorded low calcium values at least 6 months apart. We included patients of all age groups. Subsequently, their medical records were examined with special regard to demographics, etiology, the awareness of the diagnosis, the quantity and cause of hospitalizations, the length of hospital stays, the quantity and the cause of ER visits, main symptoms, prescribed therapy, calcium levels, QT intervals (cardiological indicator to measure the ventricular function), comorbidities as well as the causes of death.

Descriptive statistics included frequencies and percentages as well as mean and median for normal and abnormal distribution, respectively. Distribution was tested using Shapiro–Wilk test. In accordance with distribution, the necessary models were used to demonstrate correlations. A *P*-value of 0.05 was considered significant. All data were recorded anonymized and analyzed using Microsoft Excel®, and analysis was done using IBM SPSS Statistics Version 26®.

The main information regarding the causes of death came from death certificates and other documentation made by the healthcare staff, reporting the last hospital stay.

## Results

### Demographics of patients with at least one ER visit/hospitalization

Among 198 patients, we were able to identify 219 ER visits, 627 hospitalizations and 12 deaths during our observation period of up to 17 years (median follow up period 10,9 years (IQR 10)). The division of all patients according to their documented medical history showed that 42 (21.2%) of them did present for HP-related symptoms in the past, 107 (54.0%) patients recorded hospital/ER admissions due to different diseases (including plausible complications of chronic HP) and a group of 49 (24.7%) patients never recorded any admissions to the hospital. As expected, women took the largest share in the cohort (70.2%, *n* = 139). A postsurgical etiology with 81.3% (*n* = 161). The mean age of this cohort was 62.6 ± 18.7 years. Compared by medical history, patients who presented with acute (hypocalcemic) HP symptoms were the youngest (58.2 ± 21.8 years old). The mean age of patients who never recorded any hospital admission was 61.5 ± 18.9 years and the group of hospitalized patients was the oldest with a mean age of 64.9 ± 17.0 years (Table 1).

**Table 1:** Comparison of subgroups.

	Never presented (*n* = 49)	Presentation for acute HP symptoms (*n* = 42)	Presentation for other diseases/HP comorbidities (*n* = 107)	*P*-value
Age	61.5 ± 18.9	58.2 ± 21.8	64.9 ± 17.0	0.132
Gender	34/69.4% (female) 15/30.6% (male)	26/61.9% (female) 16/38.1% (male)	79/73.8% (female) 28/26.2% (male)	0.379
Postsurgical	41/83.70%	29/69.0%	91/85.0%	<0.001
Nonsurgical	7/14.3%	13/31.0%	10/9.3%	0.407
Unknown	1/2.0%	0/0%	6/5.7%	0.059
Standard therapy	43/87.8%	34/81.0%	96/89.7%	<0.001
PTH replacement	5/10.2%	6/14.3%	4/3.7%	0.549
No therapy/unknown	1/2.0%	2/4.8%	7/5.6%	0.045
Duration of disease	19.1 ± 17.5	21.6 ± 15.3	22.7 ± 15.0	<0.001

Patients who presented for acute HP symptoms in ER/H (*n* = 42) may have also presented for other diseases over time (‘unknown’ appears to primarily reflect patients with postsurgical HP when the surgical procedure was performed before 2005).

The group, who recorded any type of hospital admissions, consisted of 149 patients. Their mean age was 63.0 ± 18.7 years (range between 10 and 97 years). Most patients in this cohort were women (70.5%; *n* = 105), and 44 (29.5%) were men. About 87.2% (*n* = 130) required standard medication, whereas 6.7% (*n* = 10) had rhPTH_1–84_ prescribed. The remaining 6.0% did not take permanent HP medication. The mean duration of disease in this cohort was 22.4 ± 15.0 years.

In the postsurgical subgroup, we counted 92 women (73.0%) and 34 men (27.0%) men, with a mean age of 66.9 ± 15.5 years (Table 2). Patients with a nonsurgical etiology showed a larger age distribution, were younger (mean age: 41.7 ± 20.4) and had a more balanced gender distribution: 56.5% (*n* = 13) were female and 43.5% (*n* = 10) were male.

**Table 2:** Baseline characteristics of 149 patients (at least once hospitalized between 2005 and 2022).

	Postsurgical (*n* = 126)	Nonsurgical (*n* = 23)	*P*-value	Total cohort (including unknown) (*n* = 149)
Age	66.9 ± 15	41.7 ± 20.4	<0.001	63.0 ± 18.7
Gender	92/73.0% (female) 34/27.0% (male)	13/56.5% (female) 10/43.5% (male)	<0.001 <0.001	105/70.5% (female) 44/29.5% (male)
Standard therapy	112/88.9%	18/78.3%	<0.001	130/87.2%
PTH replacement	6/4.8%	4/17.4%	0.197	10/6.7%
No therapy/unknown	8/6.3%	1/4.3%	0.011	9/6.0%
Duration of disease (years)	22.6 ± 15.0	–	<0.001	22.4 ± 15.0
Number of ER visits (rate/patient)	196 (1.6)	23 (1)	<0.001	219 (1.5)
Number of hospital admissions (rate/patient)	545 (4.3)	82 (3.6)	<0.001	627 (4.2)

‘Unknown’ appears to primarily reflect patients with postsurgical HP when the surgical procedure was performed before 2005.

The exact onset of disease in the nonsurgical group could not be verified for the majority of patients (heterogeneous etiology). A calculation of the duration of disease is therefore not available.

### ER visits

About 11.9% (26 out of 219) of them happened likely due to HP, primarily due to hypocalcemia. These 26 ER visits were caused by 17 patients. Eleven of them (64.7%) visited the ER once, five patients (29.4%) visited twice, and one patient (5.9%) visited five times due to HP. Hallmark HP symptoms like paresthesia, muscle spasms and shortness of breath were documented in cases of normal or decreased calcium levels. Seizures and vertigo (*n* = 6, 7.3%) were only documented in cases of severe hypocalcemia (ionized Ca^2+^: <0.8 mmol/L or total Ca^2+^: <1.6 mmol/L). Gastrointestinal symptoms like nausea, abdominal pain or diarrhea were frequent and occurred with normal, increased or decreased calcium levels. The remaining 193 ER visits (88.1%) were unlikely related to HP, which attributed to a group of 65 patients.

### Hospitalizations

Six hundred and twenty-seven hospitalizations > 24 h were identified in 130 patients. Only 7.0% (*n* = 44, 33 patients) of those admissions were directly attributable to classical symptoms of chronic HP. The reason for HP-related hospitalizations were mainly due to continuous intravenous calcium substitution, the first introduction of HP therapy or extended diagnostics (e.g. repeated blood and urinary samples, imaging and genetic testing). Twenty-five of these patients (75.6%) were hospitalized once, six (18.2%) were hospitalized twice, one patient (3.0%) was hospitalized three times and one other patient (3.0%) was hospitalized four times due to chronic HP. The maximum number of admissions was 31, which was recorded in one patient who had a long history of CKD (rapid progressive glomerulonephritis and kidney transplantation) and multiple comorbidities.

Other diseases (e.g. CKD, fractures and cardiovascular events) were responsible for the remaining 93.0% (*n* = 583) of the hospitalizations in this cohort, which affected 96 patients (15) (Table 2). In almost half of these hospitalizations, a decreased calcium level was documented.

**Figure 2:**
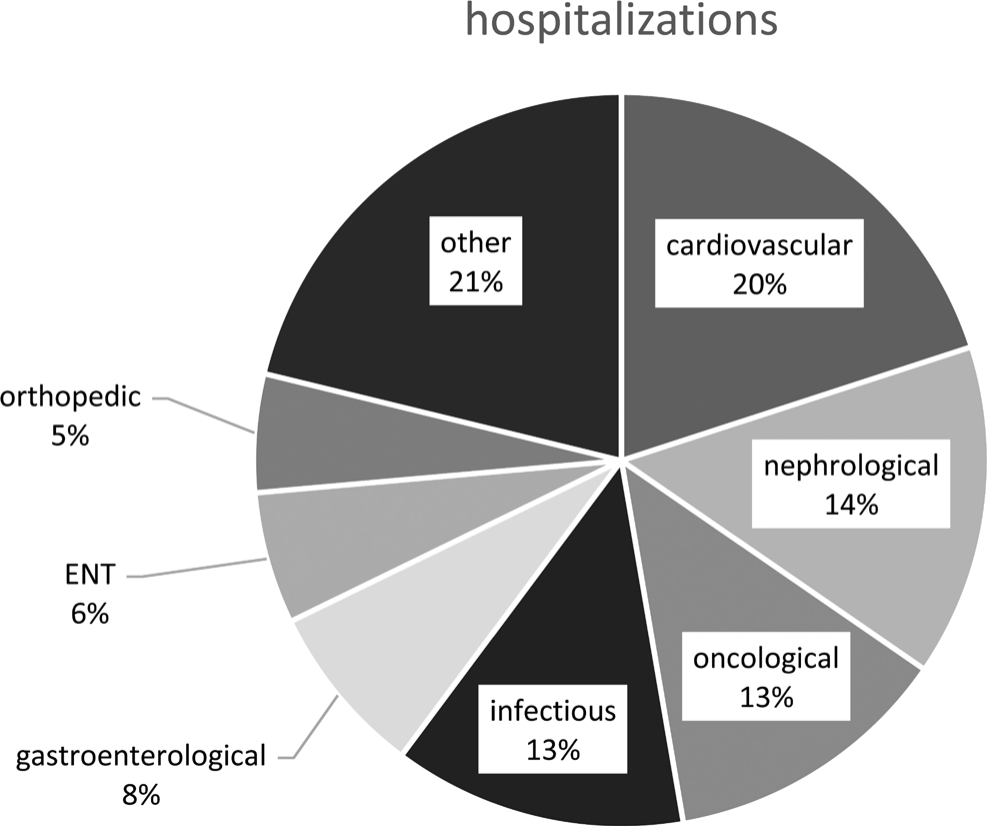
Hospitalizations subdivided by medical specialty.

These remaining 583 hospitalizations were categorized by medical departments (Fig. 2). Frequent discharge diagnoses included kidney transplantations (*n* = 13), acute renal failure (*n* = 9), nephrolithiasis (*n* = 6) as well as bone fractures (*n* = 5).

Across all etiologies, the length of hospital stay was on average 10.5 ± 13.2 days.

### Awareness

**Figure 3:**
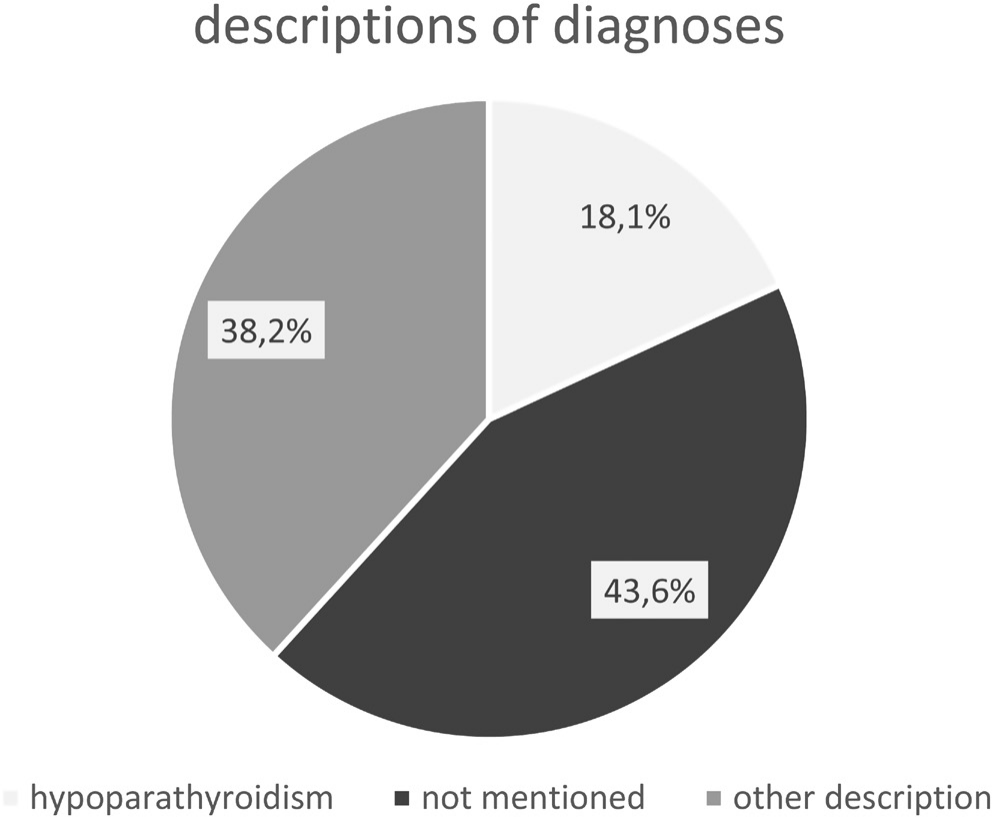
Awareness for HP – diagnosis description (hospitalized patients).

The diagnosis ‘hypoparathyroidism’ was often completely absent (44%) or not clearly stated in discharge letters (38%), using terms like ‘previous thyroidectomy’, ‘tetany’ or ‘hypocalcemia’. Overall, 48 different diagnostic terms were used. Figure 3 visualizes the variability of used descriptions. Calcium levels were ordered in 71% of all hospital admissions.

### Organ transplantation

We identified 13 patients who had received at least one kidney transplantation – seven women (53.8%) and six men (46.2%) (Table 3). In all patients, the etiology for HP was postsurgical, and the duration of disease was 27.1 ± 16.1 years. The cause for kidney transplantation was due to severe degenerative kidney disease and not HP or its therapy – all patients suffered from CKD prior to receiving the HP diagnosis (including different types of glomerulonephritis, cystic kidney disease, diabetes, Alport syndrome, nephrocalcinosis associated with hyperparathyroidism and lupus nephritis). Eight patients (61.5%) had undergone subtotal parathyroidectomy for tertiary hyperparathyroidism, which later resulted in HP (16). These patients contributed to a large proportion of hospitalizations (on average 10.8 per patient), which were not HP related but a result of their severe CKD.

**Table 3:** Baseline characteristics of patients with severe kidney disease.

	Dialysis (*n* =6)	Kidney transplant (*n* =13)
Age	69.5 ± 21.8	66.2 ± 15.0
Gender	3/50% (female) 3/50% (male)	7/53.8% (female) 6/46.2% (male)
Postsurgical	6/100%	13/100%
Reason for surgery (*n*)	(Renal) hyperparathyroidism (4), benign goiter (1) and thyroid cancer (1)	(Renal) hyperparathyroidism (8), thyroid cancer (2), benign goiter (2) and Graves (1)
Standard therapy/no therapy	6/100%	13/100%
Underlying disease (*n*)	Vascular/diabetic (4) and unknown (2)	Glomerulonephritis (2), reflux nephropathy (2), (diabetic) nephropathy (2), cystic kidneys (2), Alport syndrome (1), pyelonephritis (1), lupus (1), glomerulopathy (1) and nephrocalcinosis (1)
Number of parathyroidectomies	4/66.7%	8/61.5%
Number of ER visits (per patient), range	6 (1), 0–2	31 (2, 6), 0–10
Number of hospitalizations (per patient), range	38 (6, 3), 0–17	126 (10.5), 2–3
Death	2 (33.3%)	3 (23.1)%

One 65-year-old patient in our cohort (HP etiology unknown) received a liver transplant because of liver cirrhosis due to chronic hepatitis C infection. This patient was hospitalized seven times for the following diagnoses: reevaluation for liver transplantation, erysipelas, gastritis, fever of unknown origin following the liver transplantation, cholestasis (twice) and chronic rejection (of the transplant).

### Electrocardiogram findings – QTc time

We were able to confirm a linear and inversely proportional correlation between ionized serum calcium levels and measured QTc time by testing Pearson’s correlation coefficient (r = –0.576, *P* = 0.01) for 19 ER cases. In total, 85 QTc times were documented but only a small proportion in combination with (ionized) calcium levels. The mean QTc time in our cohort was already slightly elongated (457 ms ± 55) and reached a maximum of 609 ms in combination with severely low (ionized) serum calcium levels. No cases of ventricular arrhythmias were identified.

### Fractures

We documented the number of bone fractures and counted 53 fractures in 29 patients. Four fractures (7.5%) were the result of high-impact trauma, 16 fractures (30.2%) were the result of low-impact trauma/inadequate trauma (e.g. fall/stress fracture), 21 fractures (39.6%) were classified as osteoporotic and for 12 (22.6%) no further details were available. Subdivided by anatomical regions, the following fractures were counted: 18 (33.9%) vertebral fractures, 17 (32.1%) fractures of the lower extremity, 9 (17.0%) fractures of the upper extremity, 5 (9.4%) fractures of the face/skull, 3 (5.7%) rib fractures and one (1.9%) pelvic fracture. Twenty-three (79.3%) of these patients were hospitalized at least once and two of them (both nonsurgical etiology) were hospitalized because of HP.

### Deliveries

No deliveries or other pregnancy-associated hospitalizations or ER visits were documented in our cohort.

### Causes of death

Twelve patients died during the observation period (Table 4) – six men and six women with a mean age of 69.1 years (±11.8), ranging between 43 and 85 years. Ten (83.3%) of these patients had a postsurgical etiology and in two cases (16.7%), the etiology was not specified, but it appears likely that these were patients with neck surgery before the introduction of the electronic health record system. Three of the deceased patients had received a kidney transplantation in the past and two were on dialysis. The mortality throughout the whole cohort was 7.8%. The ultimate cause of death appeared not to be directly related to HP. Most of these patients experienced several chronic complications related to the disease (e.g. CKD, osteoporosis and depression). A relatively large number (4 out of 12) died because of infections.

**Table 4:** Causes of death in 12 patients.

Age	Etiology		Cause of death		Underlying disease	
43, female	Postsurgical		Cancer		Cancer (Peutz–Jeghers syndrome)	
71, female	Unknown		Cancer		Cancer (hepatocellular carcinoma)	
61, male	Postsurgical		Ischemic stroke + coronary heart disease + chronic obstructive pulmonary disease		CVF + kidney transplantation	
64, male	Postsurgical		Subarachnoid hemorrhage		CVF + dialysis	
67, male	Postsurgical		Chronic heart failure		Kidney transplantation	
85, female	Unknown		Myocardial infarction		CVF	
76, female	Postsurgical		Cardiac arrest		COVID-19	
61, male	Postsurgical		Sepsis (necrosis/phlegmon)		Peripheral arterial disease + dialysis	
69, male	Postsurgical		Cardiac arrest		Sepsis (pneumonia) + kidney transplantation	
81, male	Postsurgical		Bacterial tracheitis (tracheostomy)		Sepsis	
85, female	Postsurgical		Terminal cancer (mantle cell lymphoma)		Sepsis (aspiration pneumonia)	
66, female	Postsurgical		Dilated right ventricle (autoimmune hepatitis)		MOF	
**Mortality rate according to subgroup**						
Postsurgical (*n* =168)		Nonsurgical (*n* = 30)		Dialysis (*n* = 6)		Kidney transplant (*n* = 13)
7/4.2%		0/0%		2/33%		3/23%

COVID-19, coronavirus disease 2019; CVF, cardiovascular failure; MOF, multiorgan failure, including unknown.

### Patients without documented ER visits/hospitalizations

For 49 patients (24.7%), neither ER visit nor hospitalization was documented during the observation period (Table 1). This cohort resembled the rest concerning their demographic characteristics – it included 34 women (69.4%) and 15 men (30.6%). The mean age of this group was 61.5 ± 18.9 years (ranging between 26 and 91 years), and the women were slightly older (62.1 ± 19.1 years) than the men (60.0 ± 18.7 years). The postsurgical subgroup represented 83.7% (*n* = 41) of this cohort, whilst the nonsurgical represented 14.3% (*n* = 7). One person’s etiology (2.0%) remained unknown. About 87.8% (*n* = 43) received standard medication (oral calcium and/or active vitamin D) as the primary treatment, and five patients (10.2%) received rhPTH_1–34_/_1–84_. One patient (2.0%) did not require supplementation regularly. The average duration of disease was 19.1 ± 17.5 years – ranging from 2 to 64 years. None of these 49 patients required hemodialysis. Other chronic organ damages related to HP or its therapy were nephrolithiasis (*n* = 1/2.0%), hip fractures (*n* = 2/4.0%), cataract (*n* = 4/8.0%) and infections (*n* = 7/14.0%).

## Discussion

HP is the most common long-term complication following anterior neck surgery, yet it remains underdiagnosed and undertreated. These patients appear in almost all medical specialties due to acute or chronic complications. However, the burden of disease and long-term complications are usually underestimated (17, 18, 19). Only limited amount of data exist on the causes of ER visits, hospitalizations and death.

This retrospective analysis in a large tertiary care center in Austria provides detailed insight into the medical history of 198 patients over a period of 17 years. In total, we recorded 219 ER visits, 627 hospitalizations and 12 deaths. Surprisingly, patients requiring immediate treatment of hypo-/hypercalcemia-related symptoms were the minority for presentation in the hospital. Most patients presented with symptoms attributable to chronic organ damages that were not acutely related to HP, but a contributing role of HP appears possible. However, hypocalcemia was a frequent laboratory finding during hospital stays, it was presumably overlooked as follow-ups or further assessments regarding this issue were rarely mentioned in discharge letters. Patients often presented for renal/cardiovascular/oncological illness, for which HP is a known contributing factor (20, 21, 22). Prolonged QTc time was a frequent finding, but life-threatening ventricular arrhythmias were not documented. All 12 deaths appeared to be unrelated to HP at first, but comorbidities linked to HP were common.

A special subgroup consisted of 19 patients with severe CKD (13 patients after kidney transplantation and 6 with CKD stage 5 dependent on chronic dialysis). Contrary to our original assumption, CKD was not caused by chronic HP and/or its therapy in a single case. All patients in this group had postsurgical HP, mostly after subtotal parathyroidectomy for tertiary renal hyperparathyroidism. This procedure is now rarer since cinacalcet is widely used in this population, but many of these patients have a medical history over decades. This CKD group had a substantially higher rate for the endpoints ER visit/hospitalization and death. As patients with end-stage renal disease/kidney transplantation also have a higher risk for infections and cardiovascular events, reverse causality could be present in this subgroup and make the risk for the endpoints appear higher in the whole group. This is a novel finding and should prompt to further research on this topic.

A group of 49 patients (24.7%) had no documented ER visits or hospital admissions. This group showed no significant differences in baseline characteristics compared to the group with at least one ER visit/hospitalization (*n* = 149). We are unable to give a clear explanation, but besides individual factors, geographic reasons and care outside of our center may have played a role.

Given the high variability in description as well as a lack of documentation in most cases, the awareness for HP was poor – only in 20% of cases was HP documented correctly. In 80% of all patients, HP was not documented at all or only terms like ‘previous parathyroidectomy’, ‘tetany’ or ‘hypocalcemia’ were used. However, in the majority of patients, calcium levels were tested. Based on these findings, the need for improved awareness in clinical practice appears evident. The suggested emergency card would certainly be helpful (23) during hospitalizations – especially in big tertiary medical care centers. From our point of view, patients could only benefit from it by drawing attention to this illness.

The major strength of this work is the continuous collection of clinical data representing a large group of patients over a up to 17 years. An important limiting factor of our retrospective analysis is that we are unable to compare our findings to a similar or historic control group.

Moreover, not all relevant medical data could be assessed, as they were restricted to the largest tertiary care facility in the region, the Medical University of Graz. Acute presentations may have occurred in smaller regional hospitals, specialized trauma centers, a different region, or private hospitals. Also, data before 2005 could only sometimes be found in pathology reports. As such, for several patients, we were unable to define HP etiology without doubt, but it appears more likely that previous neck surgery decades ago was not referred to in more recent documents as opposed to a form of chronic nonsurgical HP.

In conclusion, we report detailed data on an Austrian cohort for almost 200 nonsurgical and postsurgical patients with HP and found surprisingly low rates of hospitalizations/ER visits directly attributable to acute symptoms of HP. In all patients, however, HP may have contributed to symptoms/acute illness.

A novel finding is the high rate of preexisting severe kidney disease in the postsurgical cohort that needs further research.

## Declaration of interest

K Amrein received speaker fees from Takeda Pharmaceutical Company Limited in the past.

## Funding

This work did not receive any specific funding agency in the public, commercial or not-for-profit sector.
